# Legal Notes for Institutional Workers

**Published:** 1915-03-20

**Authors:** 


### LEGAL NOTES FOR INSTITUTIONAL WORKERS.

### A Curious Motor-Car Case.

*Wallace v. Gilmour, Ltd., and Ward* (1915), 31 Sh. Crt. Rep. 23, a Sheriff (County) Court decision in Scotland, is one of interest to doctors more particularly in these modern days when the motor is used by the profession so extensively. The defender, Ward, was a doctor who used his own motor-car for the purposes of his own professional work. He drove the car himself, but employed a boy to accompany him to stand by the car while he was visiting patients, and to wash the car when the day's work was done. For these purposes he hired a boy from the defenders, Gilmour, Ltd. The doctor had no concern with the personality or selection of the boy. Gilmours might have sent any boy, though, as a matter of fact, they always sent the same boy, one Campbell, who was an apprentice in their service. Campbell went to their works at six in the morning. About eight he went to the garage where the doctor's motor was kept, and got it ready for the day's work. The doctor joined him at the garage, and they started on a round of visits, usually finishing about one o'clock. The boy then took the car back to the garage, washed it, and thereafter went back to his work at Gilmours' and finished the day's work there. One day after the doctor had finished his round of visits, about the usual hour, he left the car at his own house and then the boy took it to the garage to wash it. He washed it, but instead of then locking it up as usual and as he should do, he took it out in order to go home for his own dinner, and while on this trip of his own he ran on to the pavement and knocked down and injured the pursuer.

### The Court's Decision.

As the title of the action shows, the injured party sued both the garage company and the doctor. Obviously there was fault somewhere, inferring liability for damages to the person injured; but who was liable? It all depended on two points—whose servant was the boy who was driving the car, and was he when the accident occurred acting within the scope of his employment, so as to entail liability on his employer? The Court found, without hesitation, that there was no liability on the doctor. The boy was not engaged or paid by him, and even though he had been, the occurrence took place at a time when the doctor was finished with him. Then as to the other defenders, they were not liable either; for the boy's duties to them were to watch and wash Dr. Ward's car. He had finished these duties on that particular day, and proceeded afterwards on an errand of his own with the car. In doing so he was acting in a way entirely unconnected with his employment as a servant of Gilmours, and they were not responsible for his doings while so engaged. The pursuer was accordingly found to have a remedy only against the actual committer of the wrong—namely, the boy Campbell—an empty remedy, as presumably his financial position will not make him worth suing for damages; hence the endeavour to make either the doctor or the company liable.

### Doctors and the Duplication of Insurance Prescriptions.

We propose next to notice a case under the National Health Insurance Act which affects the interests of doctors—*Rex v. County of London Insurance Committee; Ex parte Salter*, 30 T. L. R. 607. Dr. Salter made an agreement with the Committee, one of the clauses of which was that,—" the practitioner shall order in the form provided such drugs and prescribed appliances as are requisite for the treatment of any patient." In pursuance of this clause the Committee provided Dr. Salter with books of forms for medical prescriptions containing carbon sheets, so that orders for drugs might be given in duplicate. The duplicate forms were for the convenience of the chemist, who might keep a copy of the prescription. Dr. Salter refused to write the prescriptions in duplicate, maintaining that to do so was entirely outside any obligation laid on him by the Insurance Act, and that it was unreasonable to ask him to act as clerk to the chemist. A few months later the Committee intimated that they proposed to vary the terms of agreements with doctors, and subsequently they sent a form of agreement to Dr. Salter which provided *inter alia*—" the practitioner shall order in duplicate on the form provided," etc. Dr. Salter struck out the words "in duplicate" and signed the agreement, returning it to the Committee with the observation that if they would undertake to provide him with duplicating books, the use of which involved no more labour or expenditure of time than was caused by the writing of single slips, he was prepared to supply prescriptions in duplicate. The next step was that the Committee dropped Dr. Salter's name from the revised list of the panel, and he then took proceedings for reinstatement.

The question turned on the construction of Section 15 (2) (6) of the Act, which confers on any duly qualified medical practitioner who so desires the right to be included in the panel list, but subject to removal when "the Insurance Commissioners after such inquiry as may be prescribed are satisfied that his continuance in the list would be prejudicial to the efficiency of the medical service of the insured." Now, Dr. Salter's name had been removed from the list without inquiry, and by his agreement he had not prejudiced in any way the right to one. The Court found that the Committee had no power to remove him for declining to make prescriptions in duplicate, for when a doctor was put on the panel list, he must remain on it either until he withdrew or until he was removed for good cause after an inquiry. So that if his refusal were wrong, the proper procedure for the Committee to take was to hold an inquiry as to whether the doctor's refusal was, in the words of the Act, "prejudicial to the efficiency of the medical service."

